# Zinc and Type 2 Diabetes: A Systematic Review with a Narrative Synthesis of Their Bidirectional Relationship and Clinical Perspectives for Personalized Nutritional Support

**DOI:** 10.3390/diseases13120396

**Published:** 2025-12-11

**Authors:** Evgeniya Klein, Daria Velina, Sherzodkhon Mutallibzoda, Svetlana Tefikova, Olga Orlovtseva, Alexander N. Kosenkov, Dmitry Kulikov, Igor Nikitin

**Affiliations:** 1 Department of Food Technology and Bioengineering, Plekhanov Russian University of Economics, 115054 Moscow, Russia; kattim67@gmail.com (D.V.); mutallibzoda.s@rea.ru (S.M.); tefikova.sn@rea.ru (S.T.); orlovtseva.oa@rea.ru (O.O.); 2 Department of Hospital Surgery, I.M. Sechenov First Moscow State Medical University, 119435 Moscow, Russia; alenkos@rambler.ru; 3 Rogov Institute of Applied Biotechnology and Food Engineering, Federal State Budgetary Educational Institution of Higher Education Russian Biotechnological University, 125080 Moscow, Russia; kulikovda@mgupp.ru

**Keywords:** nutritional support, type 2 diabetes mellitus, personalized nutrition, risk factors, zinc imbalance, zinc supplements

## Abstract

Background: Type 2 diabetes mellitus (T2DM) remains one of the most significant public health problems, and its incidence rate is steadily increasing worldwide despite scientific and technological progress in the field of medicine. The focus of research in this area is gradually shifting from classic risk factors—such as obesity, sedentary lifestyle and genetic predisposition—toward additional, potentially modifiable contributors such as micronutrient imbalances; among them are disturbances in zinc homeostasis that may influence glucose metabolism and oxidative stress. Objective: This systematic review with narrative synthesis aims to examine the bidirectional relationship between zinc status and T2DM and to evaluate whether zinc screening and personalized nutritional support could contribute to comprehensive metabolic management. Methods: A literature search was conducted in the PubMed database and the Cochrane library for studies published between 2010 and 2024. Studies assessing zinc status or supplementation in relation to the risk, progression, or management of T2DM were included. Data were synthesized narratively, focusing on clinical and mechanistic evidence. Results: Thirty studies met the inclusion criteria. Evidence indicates that zinc imbalance (both deficiency and excess) is associated with T2DM risk and outcomes. Zinc deficiency may impair insulin synthesis and signaling, promote oxidative stress and inflammation, while excessive zinc intake may induce metabolic disturbances. T2DM itself may lead to reduced zinc status via altered absorption and increased excretion. While some studies suggest modest improvements in glycemic or lipid parameters following zinc supplementation, findings remain inconsistent and context-dependent. The prevalence of suboptimal zinc status in certain populations supports the rationale for targeted screening rather than routine supplementation. Conclusions: Zinc is mechanistically involved in insulin synthesis, antioxidant defense, and inflammation control, but current clinical evidence does not justify its use as a therapeutic agent in T2DM. Instead, assessment of zinc status and individualized correction of deficiency may represent a component of personalized nutritional support, particularly for patients with long disease duration, poor dietary quality, or genetic predispositions affecting zinc metabolism.

## 1. Introduction

The first quarter of the 21st century has witnessed major breakthroughs in key areas of biology, contributing to steady improvements in life expectancy and quality of life in both developed and developing countries. Despite significant advances in science and technology, the medical community still lacks sufficiently effective preventive and therapeutic strategies to address the complex and multifactorial nature of type 2 diabetes mellitus (T2DM). The global spread of the disease is outpacing all previous projections: in 2017, the number of people living with T2DM was estimated at approximately 425 million, with an expected increase to 628.6 million by 2045 [1]. However, by 2022, updated data indicated that more than 780 million individuals were already affected [2]. Currently, nearly one in ten people worldwide is diagnosed with T2DM, and in some countries, the prevalence exceeds the global average—for example, it is over 17% in Saudi Arabia [3] and approximately 13% in China [4].

T2DM remains a major global health threat, associated with a wide range of serious socioeconomic and medical consequences. In addition to its inherent risks, T2DM significantly increases the likelihood of developing cardiovascular diseases (CVDs), including coronary artery disease, myocardial infarction, and stroke. Recent studies have also shown that T2DM correlates with adverse structural changes in the heart muscle, which requires additional monitoring of patients to prevent complications at the cardiovascular level [5]. Particularly alarming is the growing body of evidence linking T2DM to cognitive decline. The development and progression of cognitive impairment in patients with T2DM are believed to result from several interrelated mechanisms: chronic neuronal damage caused by advanced glycation end-products under hyperglycemic conditions, reduced cerebral oxygenation due to microvascular damage, impaired neuroplasticity in the setting of systemic inflammation, and disrupted synaptic transmission in the insulin-resistant brain [6]. Given the persistent lack of effective treatments for neurodegenerative diseases, the cognitive burden associated with T2DM should be recognized as a critical concern. This highlights the importance of timely prevention and the implementation of comprehensive, long-term management strategies that include both pharmacological and non-pharmacological components.

Among the widely accepted non-pharmacological approaches to managing the risks associated with T2DM, dietary interventions hold a particularly important role, as the etiology of the disease is closely linked to patients’ lifestyle and nutritional habits. Low-calorie, high-protein diets, as well as the Mediterranean diet [7]—a nutritional pattern rich in polyunsaturated fatty acids (PUFAs) and dietary fiber and low in added sugars and refined carbohydrates—have been shown to positively influence glucose metabolism. Targeted nutritional strategies also include the supplementation of specific micronutrients. For example, vitamin D supplementation has been shown to reduce local inflammation in pancreatic β-cells and improve tissue insulin sensitivity [8]; magnesium deficiency has been associated with the development of insulin resistance [9]; and the intake of dietary chromium has demonstrated beneficial effects on glycemic control [10]. Considerable attention in the literature is also given to the relationship between T2DM and zinc, an essential microelement that is involved in more than 300 enzymatic and regulatory processes and plays a significant role in maintaining metabolic homeostasis [11].

Zinc facilitates the biosynthesis, storage, and secretion of insulin in pancreatic β-cells; regulates the activity of insulin receptors in peripheral tissues; and exerts antioxidant and anti-inflammatory effects by modulating oxidative stress pathways [12]. Zinc also supports lipid metabolism by influencing lipoprotein synthesis and fatty acid oxidation. These biological functions directly intersect with the mechanisms underlying glucose metabolism regulation and insulin sensitivity modulation. This leads to the hypothesis that disruptions in zinc balance, whether deficiency or excess, may have significant implications for the pathogenesis of T2DM [2].

Data accumulated over the past two decades have revealed complex interrelationships between zinc status and the risk of developing T2DM. Observational studies have indicated that zinc deficiency may impair insulin signaling, promote oxidative stress, and stimulate inflammatory signaling pathways, thereby increasing an individual’s susceptibility to diabetes [13]. Concurrently, research has also documented a reverse association: T2DM alters zinc homeostasis by reducing intestinal absorption and increasing urinary zinc excretion, leading to a secondary deficiency of this mineral [14]. This bidirectional relationship between zinc status and the pathophysiology of diabetes underscores the need for a thorough re-evaluation of the existing evidence.

The rationale for correcting zinc status for the prevention and management of T2DM remains a subject of debate. Intervention studies demonstrate that zinc supplementation may improve glycemic control and lipid profiles in certain populations; however, the findings are inconsistent, with some trials reporting neutral or even adverse outcomes [1].

Recent advances in nutrigenetics and metabolic phenotyping have revealed that individual responses to micronutrient interventions are determined by genetic polymorphisms (e.g., in the zinc transporter gene SLC30A8), baseline nutritional status, comorbidities, and environmental exposures [15,16]. These insights emphasize that zinc’s role in T2DM should be interpreted within a framework of individual variability, supporting a precision-nutrition perspective rather than a uniform supplementation approach.

This systematic review with narrative synthesis aims to provide a comprehensive summary of the bidirectional relationship between zinc and T2DM. Specifically, the review addresses the following aspects:

(1) assessing whether zinc imbalance (deficiency or excess) contributes to the onset or progression of T2DM;
(2) examining the impact of T2DM on zinc metabolism and homeostasis;
(3) summarizing clinical evidence on the efficacy, limitations, and safety of zinc supplementation in the context of diabetes prevention and management.

Additionally, the review discusses mechanistic foundations and explores the implications of these findings for developing personalized nutritional strategies focused on maintaining optimal zinc status according to individual metabolic and genetic profiles.

## 2. Materials and Methods

### 2.1. Review Design and Guidelines

This study was conducted as a systematic review, following the PRISMA 2020 guidelines, with a narrative synthesis of findings due to heterogeneity of study designs and outcomes.

A review protocol was developed in advance and retrospectively registered in the Open Science Framework (OSF; DOI: 10.17605/OSF.IO/56X3Y). The protocol details eligibility criteria (PICOS), information sources, and analytical methods. The retrospective registration was performed as the data extraction phase had been completed prior to OSF submission.

### 2.2. Eligibility Criteria

Studies were included in this review if they met the following criteria:

- Investigated the relationship between zinc status in the body or zinc intake (including dietary supplements) and the risk, progression, or clinical outcomes of T2DM;
- Were original human studies (observational or interventional), narrative/systematic reviews, or meta-analyses;
- Included adult participants of any, sex, or ethnicity;
- Were published between January 2010 and December 2024;
- Were available as full-text articles in English.

The following exclusion criteria were applied:

- Publications that are not original research or analytical reviews (e.g., commentaries, editorials, letters to the editor);
- Studies conducted exclusively on animals and/or in vitro;
- Studies focused on comorbidities (e.g., obesity, cardiovascular disease) without addressing key T2DM outcomes (e.g., hyperglycemia, insulin resistance);
- Studies examining only the expression and/or function of zinc transport proteins in T2DM without evaluating potential nutritional interventions;
- Studies without English full-text availability.

For transparency and reproducibility, inclusion and exclusion criteria were structured using the PICOS framework for each research question. This approach allowed clear separation between studies assessing zinc deficiency as a risk factor for T2DM (Table 1), alterations in zinc status as a consequence of diabetes (Table 2), and effects of zinc supplementation for the risk of T2DM (Table 3).

### 2.3. Information Sources and Search Strategy

A comprehensive literature search was performed in the electronic database PubMed and the Cochrane Library to identify studies published between January 2010 and December 2024. The time frame was chosen to ensure coverage of contemporary studies employing standardized zinc biomarkers, current diagnostic criteria for T2DM (ADA/WHO definitions), and modern interventional designs.

The Cochrane Library was additionally searched to capture relevant clinical trials, and systematic reviews that might not be indexed in PubMed.

The search query “zinc AND type 2 diabetes” was used, capturing studies on both zinc supplementation and zinc status in the context of T2DM. During the search, built-in PubMed filters were used to limit the results to studies published in English with available full text and involving adult human participants. No additional restrictions regarding study design, population characteristics, or outcomes were applied at the initial search stage.

### 2.4. Study Selection Process

The study selection process followed the PRISMA 2020 recommendations. Two reviewers (E.K. and D.V.) independently screened the titles and abstracts to identify studies that met the predefined inclusion criteria based on the PICOS framework.

Full-text articles were then retrieved for potentially eligible records and assessed independently by both reviewers. Discrepancies in study inclusion were resolved through discussion and consensus.

**Figure 1:**
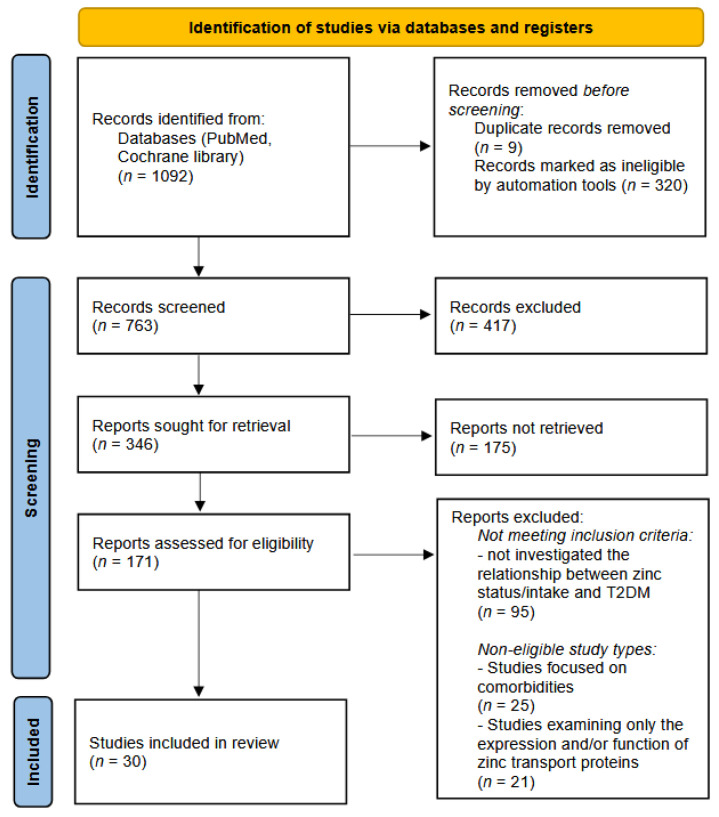
The study selection process. PRISMA flow diagram.

The overall selection process is summarized in the PRISMA flow diagram (Figure 1), showing the number of records identified, screened, excluded, and included in the final synthesis.

### 2.5. Data Collection Process

Data extraction was performed independently by two reviewers (E.K. and D.V.). The following data were collected from the included studies:

- Study design and characteristics of the participants
- Zinc status or supplementation details
- Outcome measures and results
- Study funding and conflicts of interest.

### 2.6. Risk of Bias Assessment

The methodological quality of included systematic reviews was evaluated using the AMSTAR-2 tool (16-item checklist). Each review was independently assessed by two authors (E.K. and D.V.) across all domains, including eight critical items. Ratings of ‘Yes’, ‘Partial Yes’, or ‘No’ were assigned, and overall confidence in the results was determined following AMSTAR-2 guidance.

Observational studies (cohort, case–control, and cross-sectional designs) were assessed using the Newcastle–Ottawa Scale (NOS). The NOS assessed three domains: selection (4 points), comparability (2 points), and outcome/exposure (3 points), with a maximum total score of 9. Studies scoring 7–9 were considered good quality, 5–6 moderate, and ≤4 poor.

Randomized controlled trials were assessed for risk of bias using the Cochrane Risk of Bias 2.0 (RoB 2) tool. Five domains were evaluated: randomization process, deviations from intended interventions, missing outcome data, measurement of outcomes, and selection of the reported result. Each domain was rated as low risk, some concerns, or high risk, and overall judgment followed the Cochrane guidelines.

Narrative reviews included in this synthesis were critically appraised using a custom six-domain checklist adapted from the SANRA (Scale for the Assessment of Narrative Review Articles) and AMSTAR-Lite frameworks. The domains assessed were (1) clarity of objectives, (2) comprehensiveness of literature coverage, (3) balance and control of bias in interpretation, (4) mechanistic insight or conceptual depth, (5) transparency of methodology (information sources and selection criteria), and (6) consistency with current evidence. Each criterion was rated as ‘✓✓’ (fully met), ‘✓’ (partially met), or ‘X’ (not met). Overall quality was categorized as High (≥5 fully met), Moderate (3–4 fully met), or Low (≤2 fully met).

Two reviewers (E.K. and D.V.) independently performed the assessments; disagreements were resolved through discussion. The results of the assessment are summarized in the Supplementary Materials (Table S1).

### 2.7. Data Synthesis

Due to the heterogeneity of study designs and outcome measures, the data were synthesized narratively. Studies were grouped thematically into three categories:

- Zinc imbalance as a risk factor for T2DM;
- Zinc status alterations as a consequence of T2DM;
- The role of zinc supplementation in the prevention and management of T2DM.

The study selection process is summarized in the PRISMA flow diagram (Figure 1).

## 3. Results

The process of study selection is summarized in the PRISMA flow diagram (Figure 1). A total of 907 records were identified through the PubMed database and another 185 records were found in the Cochrane library. Before screening, 320 records were automatically excluded by the PubMed filtering tools (non-English, non-human, or unavailable full-text publications). Also, 9 publications turned out to be duplicates. As a result, 763 records remained for screening by title and abstract. Of these, 417 records were excluded based on relevance. Subsequently, 346 full-text articles were sought for retrieval. However, 175 reports could not be retrieved in full text and were therefore excluded. In total, 171 full-text articles were assessed for eligibility. Following full-text review, 95 articles were excluded for not meeting the inclusion criteria, and 46 were excluded due to ineligible study types. Ultimately, 30 studies were included in the systematic review.

Of the 30 included studies, 14 examined the relationship between zinc status and T2DM, while 18 explored the potential of zinc supplementation as a preventive and/or therapeutic approach. Two publications thus combine data on both zinc status and the role of its intake in counteracting T2DM.

### 3.1. Zinc Imbalance Increases Risk of Type 2 Diabetes

Among the publications selected for this review, nine studies [1,14,17,18,19,20,21,22,23] provide evidence suggesting that inadequate zinc status is a risk factor for the development of T2DM. A summary of these studies is presented in Table 4.

As summarized in Table 4, three case–control studies [17,18,19], one cohort study [22], two systematic reviews [1,23], and three non-systematic narrative reviews [14,20,21] were included in this section.

The case–control studies were evaluated using the Newcastle–Ottawa Scale (NOS) and were all rated as good quality, each receiving 7 out of 9 points (see Table S1). All three studies were funded by non-commercial, education-oriented organizations, suggesting the absence of bias related to financial conflicts of interest. Interestingly, across all three studies, serum zinc levels in the case and control groups differed by a consistent magnitude of approximately 8–10%, which may indicate a degree of reproducibility of findings across distinct ethnic populations.

The cohort study also demonstrated good methodological quality according to the NOS, with a total score of 8 points. The study had a follow-up period of 20 years, during which dietary zinc and copper intake were assessed using a validated food-frequency questionnaire. This methodological approach, however, has inherent limitations due to self-reported dietary data, including potential underreporting or recall errors, and the inability to accurately estimate the amount of zinc absorbed from the diet. Nevertheless, the large sample size, extended observation period, and the lack of commercial influence strengthen the reliability of this study within the context of the present review.

The systematic reviews were assessed using the AMSTAR-2 checklist and were considered high quality, as all critical domains were fully satisfied (see Table S1). These reviews covered a large number of participants from various countries, with age ranges spanning from 33 to 84 years [23] and 18 to 66 years [1]. Zinc status in the included studies was evaluated both by biochemical biomarkers and by dietary intake. Interestingly, both systematic reviews also noted that excessively high zinc levels may be associated with an increased risk of type 2 diabetes, emphasizing the importance of maintaining an optimal—rather than maximal—zinc status. Importantly, both review authors explicitly addressed the potential bias of primary studies when interpreting the results, which supports the validity and robustness of their conclusions.

In contrast, the non-systematic narrative reviews were rated as low quality due to the absence of clear methodological descriptions, lack of stated objectives, one-sided interpretation of evidence, and insufficient conceptual depth. Nevertheless, these papers provided valuable mechanistic insights into potential pathways linking zinc deficiency with the development of type 2 diabetes, which will be discussed in the following section.

### 3.2. Type 2 Diabetes Causes Zinc Imbalance

Seven publications address alterations in zinc status as a pathological consequence of T2DM. These studies are summarized in Table 5.

As summarized in Table 5, this section includes one case–control study [19], one cross-sectional study [27], three non-systematic narrative reviews [14,24,26], one systematic review [25], and one umbrella review [15].

The case–control study [19] was previously discussed in Section 3.1, as it provides evidence supporting the bidirectional relationship between zinc and T2DM. Individuals with diabetes had significantly lower serum zinc levels compared with controls, while within the T2DM group an inverse correlation was observed between serum zinc and indices of insulin resistance. In other words, regardless of the underlying cause of diabetes onset, zinc status tends to deteriorate in affected individuals, yet higher zinc levels within this group are associated with better insulin sensitivity.

The cross-sectional study [27] was characterized by a heterogeneous sample and a notable imbalance in the number of participants between the case and control groups, as well as insufficient reporting on response and participation rates. Consequently, it received a moderate quality rating on the NOS (see Table S1). Nevertheless, the study provided valuable data by differentiating zinc and copper levels among patients with various diabetic complications, such as nephropathy, retinopathy, and peripheral neuropathy, which is of additional interest for study within the framework of the current review.

Among the non-systematic narrative reviews, two [14,26] were rated as low quality for reasons similar to those discussed earlier—namely, lack of methodological transparency, undefined search criteria, and absence of stated objectives. One review [24] was rated as moderate quality, as it clearly stated its aim, partially described the literature search process, and identified the databases consulted. However, it provided limited coverage of mechanistic evidence, preventing a higher overall rating (see Table S1). The reviews [14,26] mainly described potential mechanisms underlying zinc depletion under hyperglycemic conditions, whereas review [24] complemented these findings with insights on intracellular zinc content in pancreatic cells, briefly highlighting—though not fully exploring—a key mechanistic aspect of T2DM pathogenesis.

The umbrella review [15], while methodologically strong, did not contribute new evidence on zinc status in patients with T2DM but confirmed the consistent finding of excessive urinary zinc excretion secondary to impaired glucose metabolism. The systematic review [25], also rated as high quality within this work, yielded a particularly important conclusion: zinc deficiency in individuals with T2DM is not attributable to altered dietary patterns, thereby reinforcing the focus on internal metabolic dysregulation as the primary cause of zinc imbalance in diabetes.

### 3.3. Zinc Supplementation and Type 2 Diabetes: Current Evidence and Clinical Relevance

Given the complex interplay between zinc homeostasis and glucose metabolism, zinc supplementation has been explored as an adjunct to standard diabetes care. However, the clinical significance of such interventions remains uncertain, as results across randomized controlled trials vary widely depending on population, baseline zinc status, and study design. Thirteen of the publications included in the current review address this area of research. These studies are summarized in Table 6.

Table 6 summarizes ten RCTs [32,34,35,36,37,38,39,40,41,42], six systematic reviews [16,28,29,30,31,33], and two non-systematic reviews [24,26]. These studies provide up-to-date evidence regarding the rationale for zinc supplementation in various forms (including sulfate, gluconate, acetate, and oxide), as well as in combination with other nutrients and bioactive compounds (such as magnesium, chromium, vitamins A and E, and curcumin) for the treatment of T2DM and associated metabolic disorders. The main outcome measures assessed in these studies included glycemic and insulin-resistance parameters, lipid profile components, and anthropometric indicators (BMI). At the same time, studies focusing on zinc-containing proteins and inflammatory biomarkers were not included in this review, although these factors are indirectly related to the pathogenesis of T2DM.

The non-systematic reviews included in this subsection are repeated from the previous section, reflecting the authors’ intention to examine the role of zinc in T2DM from multiple perspectives, albeit in a rather superficial manner. Of particular relevance to the present work is publication [26], which—despite receiving a low-quality rating according to formal criteria—raises an important issue of genetic predisposition to T2DM in individuals carrying SLC30A8 polymorphisms. This topic is further developed in the systematic review [16], which received a moderate quality rating due to the absence of explicit statements about double data extraction and peer verification.

Systematic reviews [30,33] also received moderate ratings: the first due to the lack of double data extraction and peer review, and the second because of insufficient interpretative work regarding heterogeneous data. Nevertheless, both reviews provide valuable insights for the present analysis: the findings of [30] support the choice of appropriate dosages and duration of zinc interventions, whereas [33] demonstrates the limited relevance of such supplementation in developed countries.

Among the high-quality systematic reviews, the following are of greatest interest: review [31], which analyzes zinc efficacy depending on supplement form; review [34], which confirms the lack of justification for zinc supplementation in developed countries; and review [28], which highlights the potential beneficial effects of supplemental zinc on glycemic control and insulin sensitivity.

Among the RCTs, studies [38,41] received low quality ratings—the first due to the absence of a published trial protocol, and the second due to substantial participant dropout by the end of the intervention. Study [38] reported positive effects of combined metformin and zinc supplementation on glycemic and lipid parameters, while [41] demonstrated beneficial outcomes of zinc supplementation alone, although the specific supplement form was not indicated.

Three RCTs [35,39,40] received moderate quality ratings (“some concerns”). The first lacked sufficient information on participant adherence; the second reported an imbalance between intervention and control group sizes due to randomization errors; and the third provided an incomplete description of randomization procedures. All three studies showed positive outcomes, confirming zinc’s ability—alone or in combination with other agents—to reduce glucose levels, insulin resistance, and body weight, as well as to improve lipid profiles.

High-quality RCTs (low risk of bias) reported the following findings: study [32] demonstrated multifactorial benefits of combined zinc and magnesium supplementation; study [34] found no effect of zinc supplementation in an Australian population; study [36] showed no significant effects of multicomponent zinc-containing supplements; study [37] revealed improvements in lipid profile but not in glycemic parameters following the intake of organic zinc compounds; and study [42] reported positive effects of combined zinc and curcumin supplementation.

## 4. Discussion

### 4.1. Key Points of the Selected Publications

#### 4.1.1. Interpreting the Directionality: Can Zinc Dysregulation Precede and Predict T2DM Onset?

The very fact that an imbalance of any micronutrient leads to a disruption of the normal course of biochemical processes in the body stimulates researchers to identify specific cause-and-effect relationships such as “deficiency-disease” or “excess-disease”. In relation to zinc imbalance, such links were established through the study of the key biomarkers of T2DM, including fasting plasma glucose, glycated hemoglobin (HbA1c) and the insulin resistance index HOMA-IR (Homeostasis Model Assessment of Insulin Resistance). For example, a study of micronutrient status in postmenopausal women with T2DM demonstrated an inverse relationship between serum zinc levels and both fasting plasma glucose and HbA1c [17]. The authors attribute this effect to improved cellular insulin sensitivity, mediated by zinc-induced activation of the enzymes PI3K (phosphatidylinositol-3-kinase) and Akt (protein kinase B), along with inhibition of protein tyrosine phosphatases (PTPs). The PI3K/Akt signaling pathway ensures the movement of GLUT4 glucose transporters to cell membranes, regulates gluconeogenesis and activates glycolysis, as a result of which any disturbances in this signaling pathway can lead to deterioration of glucose metabolism, including the formation of insulin resistance [43]. Zinc’s inhibitory effect on PTPs, in turn, prevents these enzymes from blocking insulin receptor function, thereby supporting effective signal transduction and maintaining insulin sensitivity at the cellular level [44].

In another study, also including postmenopausal women with prediabetes, the authors found a significant inverse association of serum zinc levels with HOMA-IR [18]. As noted earlier, this association may be explained by the ability of zinc to suppress the dephosphorylating activity of PTP enzymes. Importantly, the association of serum zinc with HOMA-IR is confirmed in samples of different sexes: the authors of a cross-sectional study involving men and women aged 54 ± 1.2 years found differences in insulin sensitivity among patients with T2DM with low and high serum zinc levels relative to the median value of 135.5 μg/dL. It was shown that relatively high zinc levels are accompanied by less hyperinsulinemia and, as a consequence, less insulin resistance, assessed using HOMA-IR [19]. The authors associated the obtained result with the direct participation of zinc in the work of β-cells of the pancreas: the formation and crystallization of insulin requires the presence of this mineral, and the formation of an insulin–zinc complex (comprising two zinc ions) is essential for subsequent hormone secretion [45].

The relationship between zinc and insulin sensitivity in muscle tissue is discussed in a review article [20], where the authors highlight zinc’s ability to exert insulin-like effects in muscle cells in response to glucose intake. Specifically, zinc mediates phosphorylation and subsequent inactivation of the GSK3β (glycogen synthase kinase 3β) enzyme, which prevents the accumulation of glucose in the form of glycogen and whose excessive activity, accordingly, is associated with an increase in blood glucose levels [13]. The authors specifically note that the Western diet is characterized by zinc deficiency, which is why residents of developed countries risk deteriorating skeletal muscle sensitivity to insulin throughout their lives, despite the fact that this tissue accounts for up to 80% of postprandial glucose utilization [20]. Such chronic disturbances in muscle glucose metabolism may ultimately contribute not only to the development of T2DM, but also to functional impairments in muscle tissue.

Another possible mechanism linking zinc deficiency to increased T2DM risk is proposed in a review article examining the relationship between a wide range of nutrients and the disease: the authors cite the fact that zinc is a structural element of antioxidant enzymes, and therefore insufficient intake of this mineral impairs their synthesis and reduces the body’s ability to resist oxidative stress [14]. In this context, it should be noted that reactive oxygen species (ROS) can directly participate in the development of T2DM and can also accumulate as the disease progresses. The first mechanism is based on the fact that oxidative stress in pancreatic islet cells suppresses insulin gene expression, while the second is associated with increased ROS production from protein glycation products under hyperglycemic conditions [46]. In both cases, insufficient antioxidant activity means a negative outcome in the form of direct development of the disease or the occurrence of complications associated with it.

The antioxidant role of zinc is emphasized in another literature review examining the association of micronutrients with common diseases, including T2DM. The authors cite several studies showing possible damage to pancreatic β-cells by ROS in the presence of zinc deficiency, with the risk of diabetes increasing by 17% (at least in a group of women from the United States) [21].

Of particular interest are the results of a 20-year prospective cohort study of the risks of T2DM development depending on the copper-to-zinc ratio (Cu/Zn) in the serum of initially healthy women. The results showed that low daily zinc intake (<8 mg/d) simultaneously with a high Cu/Zn ratio (>0.55) is associated with an increased risk of T2DM, while a low Cu/Zn ratio (<0.55) itself has a protective effect against the disease, which is especially clearly observed in obese women and women with a high daily zinc intake (>8 mg/d) [22]. According to the authors, the probable cause of the negative outcome in women with a Cu/Zn balance shifted towards copper is a disturbance of antioxidant mechanisms due to inadequate provision of the corresponding enzymes with mineral elements. Although zinc and copper are both involved in the activity of superoxide dismutase (SOD), a key antioxidant metalloenzyme that, among other things, helps protect against T2DM [47], a relative excess of copper impairs zinc absorption, causing the enzyme activity to decrease.

At the same time, one of the notable results of the considered cohort study was the discovery of a link between excessive zinc intake (150–450 mg/d) and a number of negative consequences, including the development of T2DM [19]. These data are confirmed in a number of other publications. In particular, a systematic review of earlier prospective cohort studies provides information that among middle-aged and elderly men, subjects with the highest serum zinc levels are at increased risk of T2DM [23]. A possible explanation for the described causal relationship is the hyperactivation of pancreatic β-cells by zinc with their subsequent damage or depletion of insulin receptors [48]. Significant consequences of systematic consumption of high doses of zinc also include oxidative stress and low-grade chronic inflammation [49], with the latter consistently associated with insulin resistance due to impaired insulin signaling during activation of the NF-κB and JNK inflammatory pathways [50].

The potentially direct relationship between zinc levels and the risk of T2DM is discussed by the authors of a meta-analysis [1], who compared two studies with opposing results: in the first study, the odds of developing T2DM were 2 times higher in subjects with high serum zinc levels compared to the control group (OR = 2.19), whereas in the second study, the association was negligible (OR = 1.08). The authors of the meta-analysis explain the discrepancy in the results of the two studies by the higher median zinc level in subjects in the first case: 799.0 μg/dL versus 764.3 μg/dL in participants in the second study. While favoring the hypothesis that elevated zinc levels may directly contribute to increased T2DM risk, the authors also emphasize the uncertainty surrounding the underlying mechanisms and suggest that genetic factors involved in zinc homeostasis may play a moderating role [1].

#### 4.1.2. Pathophysiological Pathways Linking T2DM to Secondary Zinc Deficiency

In all the studies reviewed, the effect of T2DM on serum zinc levels was unambiguous, directed towards worsening the status of the microelement. Thus, in a review of the nutritional characteristics of patients with T2DM, the section on zinc is based on the fact that deficiency of this microelement often accompanies the disease due to simultaneously increased excretion of zinc from the body and impaired absorption in the intestine [24]. This is also reported by the authors of the previously reviewed case–control study [19], who recorded lower mean zinc levels in the serum of patients with T2DM compared to the control group—139.9 ± 3.8 μg/dL versus 156.5 ± 6.1 μg/dL. Similarly, the authors associate the observed differences with difficulty in zinc absorption and its active excretion in the urine. However, it is important to note that the serum zinc concentrations in both groups fall within the established reference range for healthy individuals (70–160 μg/dL) [51]. This suggests that the condition observed in patients should not be classified as a true deficiency, but rather as a relative reduction in serum zinc levels.

The issue of impaired zinc absorption has been more thoroughly examined in the review by [14]. During digestion, endogenous zinc is secreted into the intestinal lumen via bile and pancreatic juice. Under normal conditions, this zinc is reabsorbed; however, in patients with T2DM, the reabsorption process is disrupted. As a result, these individuals not only exhibit reduced absorption of dietary zinc but also experience increased loss of endogenous zinc during digestion. The authors also report a general increase in intestinal zinc excretion. A possible explanation for this phenomenon is the upregulated expression of the zinc transporter ZIP14—which facilitates zinc transport from the bloodstream into tissue—in response to elevated glucose levels and inflammatory cytokines [52].

Important insights into the decline in zinc levels among patients with T2DM were provided by a meta-regression analysis exploring the relationship between zinc intake and zinc status in both healthy and diabetic individuals [25]. First, the analysis revealed that the difference in mean whole blood zinc concentrations between case and control groups increased with disease duration: the longer the time since T2DM diagnosis, the more pronounced the zinc deficiency. Second, the study found no significant differences in dietary zinc intake between healthy individuals and patients with T2DM, except in cases involving diabetes-related complications. This latter finding partially supports the hypothesis of a direct impact of T2DM on zinc homeostasis. However, it is important to note the limitations of conventional methods used to assess dietary zinc intake, which typically fail to account for key dietary factors that affect zinc bioavailability. These include the concurrent intake of zinc antagonists such as calcium and iron, as well as phytates—compounds known to reduce zinc absorption from approximately 21% to as low as 4–16%, depending on the molar phytate-to-zinc ratio [53]. Given that widely recommended dietary patterns for individuals with T2DM often emphasize vegetarian diets, increased consumption of whole grains and legumes as carbohydrate sources, and higher vegetable intake for fiber [54], it is likely that a substantial portion of dietary zinc is consumed alongside phytates. This, in turn, significantly reduces the efficiency of zinc absorption.

Among the potential mechanisms contributing to decreased zinc levels in patients with T2DM, a review on the effectiveness of dietary supplements in managing this disease highlights glucose intolerance and insulin resistance as key factors [15]. More specifically, elevated blood glucose impairs zinc reabsorption in the renal tubules, leading to increased urinary excretion of the micronutrient [55]. In addition, insulin resistance is often accompanied by low-grade systemic inflammation [56], which can alter the expression of the zinc transporter ZIP14, as discussed earlier.

Interestingly, T2DM leads to a decrease not only in blood zinc concentrations but also in intracellular zinc levels in various organs, as noted in a review [26] referencing one of the earliest studies on the relationship between zinc and T2DM (1938). Postmortem analysis of individuals with and without T2DM revealed a 75% reduction in pancreatic zinc levels in patients with the disease. This finding may be attributed to β-cell damage characteristic of T2DM, as these cells are primary sites of zinc localization [57], as well as to genetic polymorphisms in the ZnT8 zinc transporter gene observed in some patients. Such polymorphisms can result in the production of a less functional transporter protein, reducing the efficiency of zinc delivery to insulin granules and thereby impairing zinc accumulation and storage in the pancreas [58].

Similarly to the previously discussed pattern where zinc imbalance contributes to T2DM, studies focusing on copper–zinc interactions have identified an inverse relationship as well. An observational cross-sectional study conducted in northeastern China found that T2DM was associated with an elevated serum Cu/Zn ratio, likely resulting from disproportionate excretion of the two trace elements: patients exhibited increased urinary zinc levels but no significant rise in copper excretion [27]. The Cu/Zn ratio is also influenced by inflammation and oxidative stress. Specifically, inflammatory cytokines not only promote the translocation of zinc from the bloodstream to organs (such as the liver), but also stimulate the transcription of ceruloplasmin, a copper-binding protein that carries up to 95% of serum copper [59,60]. In turn, oxidative stress displaces zinc from albumin, causing it to migrate into tissues, while simultaneously enhancing ceruloplasmin activity. Together, these processes result in a decrease in serum zinc and an increase in serum copper concentrations [59]. Consequently, an elevated Cu/Zn ratio may not only be a downstream consequence of T2DM but also serve as a biomarker of pathological processes in otherwise healthy individuals.

#### 4.1.3. Translating Evidence into Practice: Therapeutic Potential and Considerations for Zinc Supplementation

A substantial number of randomized controlled trials (RCTs) have explored the effects of zinc supplementation on glycemic control in patients with T2DM. A systematic review that combined the results of 14 RCTs involving a total of 897 participants found that supplemental zinc in the form of gluconate or sulfate (with a median elemental zinc dose of 9.25 mg/d and a mean dose of 25.83 mg/d) was associated with modest improvements in glycemic and insulin resistance markers. After intervention periods ranging from 1.5 to 12 months, the studies reported mean reductions in fasting blood glucose by 27.68 mg/dL, HbA1c by 0.48 percentage points, and HOMA-IR by 0.49 units [28]. These findings are consistent with earlier results summarized in another review [24], which noted average reductions of 18.1 mg/dL in fasting blood glucose and 0.54 percentage points in HbA1c. While these outcomes are of potential physiological interest, they should be interpreted with caution, as methodological differences and small sample sizes limit direct comparison with pharmacological interventions such as sodium-glucose co-transporter 2 (SGLT2) inhibitors, which have well-established efficacy in glycemic management [61].

Another common focus of RCTs is the lipid profile in patients with T2DM, owing to the strong correlation between dyslipidemia and key drivers of disease progression, such as oxidative stress and impaired insulin sensitivity [62]. Several studies have demonstrated positive associations between glucose levels and both triglyceride (TG) and low-density lipoprotein (LDL) concentrations [63]. Moreover, dyslipidemia in patients with T2DM significantly increases the risk of cardiovascular complications, including myocardial infarction and stroke [64]. Therefore, improving the lipid profile should be considered a critical component of nutritional therapy for T2DM. The potential role of zinc in this context was examined in a meta-analysis [29] that synthesized data from 14 RCTs assessing the effects of zinc supplementation on lipid parameters, including LDL, TG, high-density lipoprotein (HDL), and total cholesterol (TC). The relevance of HDL and TC is underscored by clinical evidence indicating that HDL has a beneficial effect on glycemic control and β-cell function [65], while excess cholesterol exerts proinflammatory effects and damages pancreatic islet cells [66]. The meta-analysis found that supplementation with zinc sulfate or gluconate (providing 30–50 mg/d of elemental zinc) for periods ranging from 6 to 54 weeks led to reductions in LDL by 5.16–7.56 mg/dL, TG by 9.81–14.83 mg/dL, and TC by 9.34–17.48 mg/dL, along with increases in HDL by 2.31–4.48 mg/dL [29]. These outcomes can be attributed to zinc’s involvement in lipolysis—as a structural component of zinc-α2-glycoprotein—and to its regulatory role in modulating the transcription of lipid metabolism-related genes [67].

Supplementation with a combination of zinc and magnesium over a 12-week period was associated with modest improvements in key metabolic indices in patients with comorbid T2DM and coronary heart disease [32]. The regimen, consisting of 150 mg zinc sulfate and 250 mg magnesium oxide daily, reduced fasting blood glucose and elevated HDL cholesterol levels by 9.44 mg/dL and 2.09 mg/dL, respectively. Furthermore, it attenuated inflammation, as evidenced by a 0.85 mg/L reduction in C-reactive protein (CRP). The anti-inflammatory properties of zinc, which likely underpin this effect, are thought to involve the inhibition of the NF-κB signaling pathway. One proposed mechanism for this inhibition is the zinc-dependent regulation of peroxisome proliferator-activated receptor alpha (PPAR-α), a nuclear receptor with known anti-inflammatory functions [68].

When evaluating complex supplements containing zinc, it is important to determine whether the desired effect is achieved through the contribution of all biologically active components, or whether individual nutrients (in this case, zinc) do not themselves provide any beneficial properties to the overall effect of the supplement. In a RCT involving 98 patients with T2DM, the effects of consuming high doses (50,000 IU) of vitamin A and 100 mg of vitamin E, either with or without zinc, were compared. The results showed that the vitamins alone improved glycemic control (specifically, by reducing HbA1c and fasting blood glucose levels), while the combination with zinc enhanced this effect and additionally increased HDL levels [40]. Similarly, the authors of another RCT [42] demonstrated that combined intake of curcumin and zinc potentiated the effects of curcumin alone on lowering LDL and increasing HDL, whereas the reductions in TG and TC levels were, conversely, attenuated. These findings are partially consistent with those of another RCT [37], according to which the primary and most pronounced effect of zinc supplementation was the elevation of HDL levels rather than the reduction in TG or improvement in glycemic parameters.

In contrast to the aforementioned studies, trial [39] did not aim to assess the individual contribution of zinc to the overall effect of a supplement that also contained biologically active components such as inositols, α-lactalbumin, and *Gymnema sylvestre*. The authors reported that the inclusion of zinc in the supplement contributed to reductions in BMI and serum TG levels among participants; however, this statement appears to be largely hypothetical and based on previously published data. Furthermore, considering the findings from study [42] discussed above, it can be hypothesized that zinc may have attenuated the effects of the organic components on TG levels.

Particular interest is drawn to the RCT [38] that assessed the combined use of zinc with a common antidiabetic drug, metformin. According to the findings, administration of metformin together with zinc produced a more pronounced beneficial effect compared to metformin with placebo, specifically resulting in greater reductions in fasting glucose (21.52 mg/dL—Zn vs. 9.25 mg/dL—placebo) and postprandial glucose (47.53 mg/dL—Zn vs. 18.55 mg/dL—placebo), as well as HbA1c (0.79%—Zn vs. 0.41%—placebo). Moreover, at the end of the trial, the zinc group exhibited lower TG levels compared to the control group (91.70 ± 12.75 mg/dL vs. 111.30 ± 38.46 mg/dL), while the increase in HDL was not statistically significant. Taken together, the results from several RCTs [37,38,40,42] indicate a differentiated action of zinc when combined with various nutrients and pharmacological agents, suggesting the existence of potential synergistic interactions.

Several studies included in this review cast doubt on the benefits of zinc supplementation for reducing the risk of T2DM. For instance, a review of three RCTs lasting from 4 to 12 weeks described the effects of zinc supplementation on glycemic and lipid parameters as neutral [33]. Similarly, a more recent double-blind RCT with a 12-month duration found that daily supplementation with 30 mg of zinc gluconate did not produce a statistically significant effect compared to placebo. Based on these findings, the authors concluded that additional zinc supplementation may not be justified in populations from developed countries [34]. These findings are partly consistent with another study [69], which demonstrated that the beneficial effects of higher dietary zinc intake were observed more frequently among rural residents than urban ones. This discrepancy was attributed primarily to the higher prevalence of risk factors for metabolic diseases in urban populations, such as smoking, physical inactivity, and unhealthy diet. Overall, the potential impact of zinc supplementation may be limited by adverse lifestyle and environmental factors that cannot be effectively mitigated through nutritional interventions alone.

Evidence also indicates that the effects of zinc supplementation are highly variable, depending on dosage, duration, and chemical form. A meta-analysis [30] aimed to address the selection of optimal dosage and duration of nutritional intervention by comparing the outcomes of RCTs using high (≥25 mg/d) versus low (<25 mg/d) doses of zinc, as well as short-term (<12 weeks) versus long-term (≥12 weeks) interventions, in terms of their effectiveness in managing the risk of T2DM and CVDs. The analysis concluded that prolonged intake of low-dose zinc yielded the most favorable outcomes, suggesting that consistent, long-term fortification of widely consumed food products may be more effective than short-term or high-dose supplementation strategies. A meta-analysis of 32 RCTs found that inorganic forms of zinc were more effective than organic forms in improving glycemic control [31]. This result appears to contradict numerous studies demonstrating the superior bioavailability of organic zinc compounds such as bisglycinate, picolinate, and citrate, compared to inorganic forms like zinc oxide and sulfate [70]. The apparent inconsistency may be explained by differences in elemental zinc dosage: participants receiving inorganic zinc were supplemented with an average of 52 mg/d, whereas those taking organic forms received only 40 mg/d [31]. These findings suggest a dose-dependent effect of zinc supplementation, particularly within the context of short-term interventions.

Another factor that may influence the effectiveness of zinc supplementation in reducing the risk of T2DM is the presence of genetic polymorphisms affecting zinc metabolism. As noted in a review by [26], individual differences in response to nutritional interventions may depend on specific variants of the SLC30A8 gene, which encodes the zinc transporter ZnT8. This protein is predominantly expressed in pancreatic β-cells and is responsible for delivering zinc to insulin granules. Single nucleotide polymorphisms (SNPs) in this gene can impair transporter function, thereby disrupting insulin storage and secretion and increasing the risk of T2DM [71]. The potential moderating effect of zinc intake on glycemic control in individuals carrying risk variants of SLC30A8 was evaluated in a meta-analysis of 14 cohort studies. The results indicated that regular intake of 14 mg of zinc per day could mitigate the hyperglycemic effect of the rs11558471 polymorphism (A allele), reducing fasting glucose levels by 0.024 mmol/L in individuals with the A/G genotype and by 0.048 mmol/L in those with the A/A genotype [16]. However, further research is needed to determine whether zinc supplementation is similarly effective for other risk variants, such as the rs132666634 SNP (C allele), which is common in the European population and is associated with a 14% increased risk of T2DM per risk allele [71].

### 4.2. Zinc as an Element of Personalized Nutritional Support for Patients with T2DM

An analysis of current scientific evidence has identified several key factors and limitations related to the use of zinc supplements for nutritional support in patients with T2DM. This section explores the specific role zinc may have in the disease context, the patient groups that might benefit from additional zinc supplementation, the optimal forms of supplementation, and the conditions under which it should be administered.

#### 4.2.1. Mechanistic Aspects of the Role of Zinc in T2DM

Given zinc’s dual role—where deficiency can disrupt physiological processes contributing to T2DM, while excessive accumulation can precipitate symptoms of toxicity—the assessment of zinc status in patients is critically important. This evaluation should be guided by reliable biomarkers, which can range from established indicators like plasma zinc concentration to more complex measures such as zinc-binding metallothioneins or the presence of genetic polymorphisms in the SLC30A8 gene.

For patients with T2DM and impaired zinc status, normalizing serum zinc levels and achieving a moderate increase within the upper physiological limit (≤160 μg/dL [51]) may provide some beneficial effects, including the following:

(1). Improved glycemic control and insulin signaling. Zinc acts as an essential cofactor for numerous enzymes involved in glucose metabolism, including key components of the glycolytic and gluconeogenic pathways. It contributes to the structural stability of insulin and facilitates its proper storage and secretion within pancreatic β-cells via the zinc transporter ZnT8 (encoded by SLC30A8) [16,58]. By promoting autocrine insulin signaling and maintaining β-cell integrity, zinc enhances the phosphorylation of insulin receptor substrate (IRS-1) and activation of the downstream PI3K/Akt pathway, thereby improving glucose uptake by peripheral tissues [43].
(2). Modulation of lipid metabolism and body composition. Zinc plays a fundamental role in modulating lipid metabolism, acting as a key regulator of transcription factors. It activates the peroxisome proliferator-activated receptor alpha (PPAR-α), which is responsible for fatty acid oxidation and their utilization as an energy source. At the same time, zinc suppresses the activity of sterol regulatory element-binding proteins (SREBPs)—the principal inducers of cholesterol and fatty acid synthesis [68]. Furthermore, zinc is critically important for maintaining a healthy body composition. Acting as a cofactor for numerous metalloenzymes, it participates in processes of proteogenesis (protein synthesis) and lipolysis (fat breakdown). Zinc is required for the activity of key enzymes involved in amino acid turnover and muscle tissue function. Under conditions of zinc deficiency, these processes become impaired, leading to adverse changes in body morphology—specifically, a reduction in skeletal muscle mass (sarcopenia) accompanied by an increase in visceral fat [72].
(3). Enhancement of antioxidant defense mechanisms. Zinc contributes to the maintenance of redox homeostasis primarily through the activation of specific antioxidant enzymes, such as superoxide dismutase (Cu/Zn-SOD) and metallothioneins. These molecules are responsible for neutralizing ROS and regulating intracellular metal balance. Moreover, zinc exhibits protective properties toward cellular membranes by preventing lipid peroxidation. This effect is achieved through its competitive antagonism with pro-oxidant metals—iron and copper—which are capable of initiating chain reactions of oxidative damage [59,60]. Given that oxidative stress acts both as a trigger and as a consequence of the progression of T2DM [73], maintaining an adequate zinc status helps to break the vicious cycle leading to pancreatic β-cell damage and the development of insulin resistance [14,46].
(4). Regulation of inflammation and immune response. Zinc demonstrates potent anti-inflammatory effects, primarily by suppressing the NF-κB signaling cascade. Its action involves inhibiting IκB kinase activation, which in turn prevents the nuclear translocation of NF-κB and the subsequent transcription of proinflammatory cytokines such as TNF-α, IL-1β, and IL-6 [32,68]. This downregulation of cytokine production not only delays the onset of insulin resistance but also mitigates the progression of T2DM complications—including neuropathy, retinopathy, and nephropathy—by targeting the chronic inflammation that underpins them [74]. Furthermore, zinc contributes to overall immune homeostasis by enhancing thymic hormone activity and guiding T-cell differentiation, thereby reducing the systemic inflammatory burden [75].

Importantly, chronic zinc excess is associated with a range of adverse effects, including metabolic and cellular consequences such as the following:

(1). β-cell hyperactivation and insulin receptor desensitization. Sustained high zinc exposure may lead to excessive stimulation of pancreatic β-cells, resulting in compensatory hyperinsulinemia and eventual downregulation of insulin receptors on target tissues [48]. This paradoxical effect can impair insulin signaling and mimic the very metabolic disturbances zinc supplementation is intended to prevent.
(2). Induction of oxidative and inflammatory stress. Excess zinc may catalyze the overproduction of ROS in mitochondria and disrupt the balance of other essential trace elements, particularly copper and iron, which are vital for antioxidant enzyme function [76]. Moreover, elevated luminal zinc concentrations can alter gut microbiota composition by inhibiting beneficial commensal bacteria, thereby promoting low-grade inflammation and metabolic endotoxemia [49]. These mechanisms collectively contribute to oxidative injury, chronic inflammation, and potential worsening of insulin resistance.

As demonstrated, the outcomes of zinc supplementation can vary dramatically—from beneficial to potentially adverse—depending on the resulting zinc status. This underscores the critical importance of carefully monitoring serum zinc levels at the baseline, throughout the course, and upon completion of any nutritional intervention.

#### 4.2.2. Differentiation of Patients When Prescribing Zinc Supplements

As previously discussed, the effects of zinc supplementation range from neutral to clinically meaningful [24,28,32,33,34,35,36,37,38,39,40,41,42,69], largely due to interindividual variability. Several factors may influence zinc metabolism and thereby modulate the response to supplementation, including the following:

(1). Duration of T2DM and presence of complications. It is possible that a long duration of the disease reduces the effectiveness of zinc supplementation. For example, in study [36], the inclusion criteria required participants to have had T2DM for at least one year, and in this study, the zinc-enriched product did not produce statistically significant results compared to placebo. In contrast, in study [38], which involved newly diagnosed patients, zinc supplementation showed promising positive results. In patients with a long history of diabetes, persistent low-grade inflammation and oxidative stress may lead to altered zinc metabolism, decreased zinc absorption, and redistribution of zinc within tissues. This could reduce the bioavailability of supplemented zinc and diminish its antioxidant and insulin-sensitizing effects [59,60]. Regarding the complications of T2DM, important findings were reported in study [27]. Specifically, the authors showed that serum zinc levels were reduced in patients with nephropathy, retinopathy, and peripheral neuropathy compared to patients without complications. The lowest zinc status was observed in patients with retinopathy, whereas the highest urinary zinc excretion was found in those with neuropathy. Reduced serum zinc levels may reflect both increased zinc losses (as observed in nephropathy and neuropathy) and tissue redistribution of zinc due to oxidative stress and inflammation. The particularly low zinc status in patients with retinopathy may indicate a protective role of zinc against oxidative damage in retinal tissues [77].
(2). The qualitative and quantitative composition of the patient’s diet, as well as the synergistic and antagonistic interactions between zinc and other dietary components. Zinc absorption can be impaired by the presence of phytates, which bind zinc to form insoluble complexes [53]. However, this negative effect may be mitigated by the simultaneous intake of zinc and dairy products, as zinc preferentially binds to amino acids and casein phosphopeptides, facilitating its subsequent absorption by intestinal enterocytes [78]. Reduced zinc absorption has also been observed when zinc is co-administered with iron or calcium [53]; however, some studies have failed to confirm these findings [79], suggesting that the concurrent intake of these nutrients may warrant additional monitoring of zinc bioavailability.
(3). Characteristics of the area in which patients reside. There is significant differentiation in the effectiveness of zinc intake for combating T2DM between rural and urban populations [1,69], which can be attributed to a wide range of factors—from lifestyle and environmental conditions to the level of infrastructure development. For instance, scientific evidence highlights the positive impact of physical activity on glycemic control [80], alongside reports of a negative effect of aerobic exercise on serum zinc levels [81]. This suggests that the outcomes of nutritional support may be influenced by regional and environmental factors such as the predominant type of employment (e.g., agriculture, industrial labor, or sedentary office work), access to sports facilities and opportunities for physical activity (availability of infrastructure and free time), and the structure of local transportation systems (development of pedestrian areas, quality of cycling infrastructure, convenience of public transport). Additional differentiating factors between urban and rural populations may include income level, stress exposure, work schedule, smoking initiation age and duration [82]—all of which can affect biological processes and, taken together, potentially diminish the effectiveness of nutritional support.
(4). Presence of genetic predispositions to T2DM and/or disorders of zinc metabolism. The interaction between the body and the external environment is largely mediated by genetic factors. As of 2022, more than 380 single nucleotide polymorphisms (SNPs) have been identified that may influence glycemic parameters and the overall risk of developing T2DM [72]. This review discussed the outcomes of nutritional interventions in patients carrying specific polymorphisms of the SLC30A8 gene, which encodes the zinc transporter ZnT8 [16]. However, future studies should aim to take into account a broader range of gene–gene and gene–environment interactions in order to more accurately predict the effectiveness of zinc supplementation for specific population subgroups.

The efficacy of zinc supplementation in T2DM is multifactorial and depends on a combination of clinical, dietary, environmental, and genetic factors. Therefore, the use of zinc therapy requires an individualized approach that takes into account the patient’s status, diet, living conditions, and genetic characteristics.

#### 4.2.3. Choosing a Zinc Supplement Form

The choice of appropriate zinc forms and the duration of nutritional intervention must be considered, as these elements are also part of a personalized approach. In cases where short-term intervention (less than four months) is necessary for any reason, higher doses of zinc (within safe limits) may be recommended [31]. In contrast, the greatest efficacy in preventing or managing T2DM has been associated with the long-term intake of lower zinc doses (<25 mg/d) [30]. In both scenarios, the selection of a zinc formulation should take into account factors such as bioavailability, organoleptic properties, and cost. For example, zinc oxide is the most affordable and commonly used form, although it has a characteristic metallic taste; chelated zinc forms exhibit high bioavailability; and microencapsulated zinc offers the most favorable organoleptic qualities [83]. Each of these forms may be best suited to different target populations depending on specific clinical or consumer needs.

In some cases, a personalized nutritional support involves not only tailoring zinc intake parameters but also modulating the mechanisms responsible for the elimination of this micronutrient from the body. Inflammatory cytokines and elevated glucose levels are known to stimulate the translocation of zinc to the gastrointestinal and excretory systems, from where it is either not reabsorbed into the bloodstream or reabsorbed only partially [52,59]. Therefore, to enhance the overall efficacy of zinc-based nutritional support, it is advisable to align patients’ dietary patterns with anti-inflammatory and hypoglycemic principles.

In this context, the combination of zinc with other biologically active compounds within a multicomponent supplement is of particular interest. The present review identified several effective and several ineffective combinations, in particular the following:

(1). Zinc and magnesium. The combined intake of zinc and magnesium exerted a complex beneficial effect on the patients’ bodies, reducing fasting glucose levels, improving the lipid profile by increasing HDL levels, and decreasing C-reactive protein concentrations [32]. The synergistic action of these two minerals was further described in study [84] in the context of maintaining neuronal cell viability and functionality. This effect can also be extrapolated to cellular processes involved in the pathogenesis of T2DM, including impaired insulin signaling, oxidative stress, and inflammatory responses, where zinc and magnesium may jointly modulate enzyme activity and signaling pathways, thereby protecting pancreatic β-cells.
(2). Zinc with vitamins A and E. The combination of zinc with vitamins A and E also demonstrated a synergistic effect in improving glycemic control and lipid profile [40]. This effect may be explained by the close functional interrelationship between these nutrients. Specifically, zinc is required for the synthesis of retinol-binding protein (RBP), which transports vitamin A from the liver to peripheral tissues [85]; in addition, zinc maintains the sulfhydryl groups in glutathione-related proteins responsible for the regeneration of vitamin E [86]. Thus, zinc not only exerts its own protective effects against T2DM but also enhances the similar properties of vitamins A and E by supporting their functional activity in the body.
(3). Zinc and curcumin. The combination of zinc with curcumin produced inconsistent results: on one hand, a synergistic effect was observed in terms of improving HDL and LDL levels; on the other hand, the beneficial effects on TG and TC observed for each compound individually were diminished when combined [42]. Given the lack of significant influence on glycemia, the zinc + curcumin combination may be considered of limited practical relevance.
(4). Zinc in broad multicomponent formulations. The combination of zinc with a wide range of bioactive compounds may be inappropriate, since it becomes difficult to control synergistic and antagonistic interactions among the components. In study [39], doubts arose regarding zinc’s specific contribution to the overall outcome, as the authors’ interpretation was based on hypothetical data. In another study [36], no positive effect was observed despite the product containing numerous potentially beneficial components, including vitamin C, niacin, riboflavin, thiamine, cyanocobalamin, folic acid, and chromium. This may be attributed to a concealed mutual compensation of adverse and beneficial effects among the ingredients.

#### 4.2.4. Final Recommendations for Personalized Nutritional Support

The collective evidence suggests that zinc supplementation may represent a reasonable adjunctive nutritional support strategy for patients with T2DM when tailored to individual metabolic and genetic profiles. However, the optimal dosage, duration, and form of supplementation depend on baseline zinc status, genetic polymorphisms, and coexisting metabolic disturbances. Table 7 provides a concise summary of evidence-based directions for the personalized use of zinc in T2DM, integrating clinical trial outcomes with mechanistic insights discussed above.

The evidence summarized in Table 7 indicates that zinc supplementation can yield measurable metabolic benefits in individuals with T2DM when guided by objective biomarkers and individualized assessment. Correcting subclinical zinc deficiency appears to improve glycemic control, lipid metabolism, and inflammatory status; however, supplementation in patients with normal or elevated zinc levels is unlikely to provide additional benefit and may even exert adverse metabolic effects. Moreover, populations residing in developed or urban environments, where overt zinc deficiency is uncommon and the influence of other negative factors is great, are less likely to experience clinically meaningful improvements from additional zinc intake.

Collectively, these findings challenge the traditional paradigm of indiscriminate micronutrient supplementation and underscore the necessity of a precision-nutrition approach. Such an approach should integrate systematic assessment of zinc status, Cu/Zn ratio, and relevant gene–nutrient interactions (e.g., SLC30A8 polymorphisms) into clinical decision-making for T2DM management. Nevertheless, further well-designed randomized controlled trials are needed to define optimal dosing strategies, clarify the threshold between physiological benefit and toxicity, and validate the long-term safety of targeted zinc interventions. Transitioning from a “one-size-fits-all” model to biomarker-driven, individualized nutritional strategies will be essential for maximizing therapeutic efficacy, ensuring patient safety, and promoting efficient use of healthcare resources.

### 4.3. Limitations

Despite the systematic process of literature search and study selection, several important limitations of this review should be acknowledged. First, restricting the search to the PubMed database and the Cochrane library, as well as including only English-language publications with full-text availability, introduces the risk of language and publication bias, associated with the exclusion of some relevant evidence. Second, the studies included in this review are highly heterogeneous, differing in design, population characteristics, and outcomes. This variability complicates direct comparison and precludes quantitative synthesis. As a result, the conclusions of this review are necessarily descriptive and should be interpreted with caution. Third, the inclusion of meta-analyses and review articles as sources may have led to the unintentional transfer of subjective interpretations and increased the risk of double-counting evidence. Although these sources were not used as primary evidence but rather to capture broader trends and integrate findings that might otherwise be inaccessible due to language or authorship barriers, their potential influence on the overall strength of associations should not be overlooked. Finally, while the review protocol was retrospectively registered in the Open Science Framework (OSF), it was completed after the data extraction stage. Future updates of this review should consider preregistration before data collection and the inclusion of additional databases to further enhance transparency and reproducibility.

Taken together, these limitations indicate that the conclusions of the present review should be regarded as hypothesis-generating rather than definitive. To confirm and expand upon our findings, more rigorous systematic reviews and meta-analyses are required, incorporating standardized risk of bias assessment tools and focusing primarily on high-quality original studies.

## 5. Conclusions

Zinc plays an important but indirect role in the complex metabolic network underlying T2DM. Mechanistic and observational data consistently demonstrate that zinc is involved in insulin synthesis and secretion, redox balance, and inflammation control. However, current clinical evidence remains insufficient to support zinc supplementation as a standard or disease-specific therapeutic strategy for T2DM.

The findings of this review emphasize that the observed associations between zinc status and glycemic control are heterogeneous and strongly context-dependent. Differences in baseline zinc levels, dietary patterns, comorbidities, genetic polymorphisms (particularly in the SLC30A8 gene), and the form and dose of supplementation all contribute to inconsistent outcomes across studies. Therefore, broad recommendations for zinc supplementation are not currently justified.

Nonetheless, given the relatively high prevalence of marginal zinc deficiency in certain populations—especially in individuals with long-standing diabetes, suboptimal diets, or increased urinary zinc losses—routine evaluation of zinc status could be considered as part of comprehensive nutritional assessment. In such cases, individualized correction of confirmed deficiency may serve as an adjunct component of personalized nutritional support rather than a pharmacological intervention.

Future research should focus on identifying metabolic and genetic profiles that predict responsiveness to zinc correction and on establishing standardized biomarkers to define zinc status more precisely. This precision-oriented approach could clarify which patient subgroups may truly benefit from targeted nutritional support involving zinc and ensure safety by avoiding excessive intake.

In summary, zinc cannot yet be regarded as a therapeutic agent for T2DM, but it remains a relevant element in the broader framework of metabolic health and personalized nutrition.

## Figures and Tables

**Table 1:** PICO. Zinc imbalance as a risk factor for T2DM.

Element	Description	Inclusion Criteria	Exclusion Criteria
Population (P)	Healthy adults, adults with prediabetes or diagnosed with T2DM	Included adult participants of any, sex, or ethnicity	Studies conducted exclusively on animals and/or in vitro
Intervention/Exposure (I)	Low dietary zinc intake or low circulating zinc levels	Dietary, serum/plasma zinc assessment	-
Comparison (C)	Normal or high zinc status	-	-
Outcomes (O)	Incidence of T2DM; fasting glucose, HbA1c, insulin resistance indices	-	Outcomes unrelated to glucose or lipid metabolism
Study design (S)	Cohort, case–control, cross-sectional studies, reviews	Original human studies, narrative/systematic reviews, or meta-analyses	Commentaries, editorials, letters to the editor; animal studies

**Table 2:** PICO. Altered zinc homeostasis as a consequence of T2DM.

Element	Description	Inclusion Criteria	Exclusion Criteria
Population (P)	Patients with established T2DM, hyperglycemia, insulin resistance	Included adult participants of any, sex, or ethnicity with established T2DM, hyper-glycemia, insulin resistance	Type 1 diabetes, gestational diabetes
Intervention/Exposure (I)	Presence of T2DM, hyperglycemia, insulin resistance	-	Studies focused on comorbidities (e.g., obesity, cardiovascular disease) without addressing key T2DM outcomes (e.g., hyperglycemia, insulin resistance)
Comparison (C)	Healthy normoglycemic controls	-	-
Outcomes (O)	Serum/plasma zinc, urinary zinc excretion	-	Zinc-dependent enzymes
Study design (S)	Cohort, case–control, cross-sectional studies, reviews	Original human studies, narrative/systematic reviews, or meta-analyses	Commentaries, editorials, letters to the editor; animal studies

**Table 3:** PICO. Zinc supplementation and the risk of T2DM.

Element	Description	Inclusion Criteria	Exclusion Criteria
Population (P)	Healthy adults, adults with prediabetes or diagnosed with T2DM	Included adult participants of any, sex, or ethnicity	Studies conducted exclusively on animals and/or in vitro
Intervention/Exposure (I)	Zinc supplementation (oral, any form)	Oral supplementation (single or combined)	Dietary intake assessment only (no supplementation)
Comparison (C)	Placebo or no zinc supplementation	-	-
Outcomes (O)	HbA1c, fasting glucose, insulin resistance (HOMA-IR), lipids	-	Outcomes unrelated to glucose or lipid metabolism
Study design (S)	Randomized controlled trials (RCTs), reviews	Original human studies, narrative/systematic reviews, or meta-analyses	Commentaries, editorials, letters to the editor; animal studies; observational studies

**Table 4:** Characteristics of publications mentioning zinc imbalance as a risk factor for T2DM.

Author, Year	Research Type	Characteristics of the Participants	Zinc Status Details	Brief Conclusions on the Problem	Funding	Quality Assessment *
Skalnaya M.G. et al., 2017 [17]	Case–Control Study	*n* = 128 Russia, Moscow Postmenopausal women 55.8 ± 5.3 years (T2DM), 56.7 ± 6.1 years (control) 25.3 kg/m^2^ (T2DM), 25.4 kg/m^2^ (control)	Serum Zn concentrations were 8% lower than in the control group	Inverse association between serum zinc concentration and fasting plasma glucose and HbA1c levels	Supported by the Russian Ministry of Education and Science	Good
Skalnaya M.G. et al., 2018 [18]	Case–Control Study	*n* = 160 Russia, Moscow Postmenopausal women 57 ± 7 years (Prediabetes), 57 ± 6 years (control) 24.7 ± 2.8 kg/m^2^ (Prediabetes), 24.6 ± 2.1 kg/m^2^ (control)	Serum Zn concentrations were 9% lower than in the control group	Inverse association between serum zinc concentration and HOMA-IR	Supported by the Russian Ministry of Education and Science	Good
Safarzad M. et al., 2023 [19]	Case–Control Study	*n* = 112 (32 males and 80 females) Iran, Gorgan 54 ± 1.2 years (T2DM), 50.2 ± 1.7 years (control)	Serum Zn concentrations were 10.7% lower than in the control group	Inverse association between serum zinc concentration and HOMA-IR	Supported by the Golestan University of Medical Sciences	Good
Zhang X. et al., 2021 [20]	Review	-	Physiological level of plasma zinc (20 μmol/L) exerted an insulin-mimetic effect	Zinc deficiency impairs skeletal muscle insulin sensitivity	No funding	Low
Siddiqui K. et al., 2014 [14]	Review	-	Low zinc serum/plasma levels (lower than 84–159 μg/dL) lead to poor or slowed wound healing and impair resistance to oxidative stress	Zinc deficiency increases the risk of T2DM due to decreased activity of antioxidant enzymes	No funding	Low
Sumaily K.M., 2022 [21]	Review	*n* = 82,000 United States Women	Low zinc intake was associated with a 17% higher risk of developing diabetes compared with adequate intake	Zinc deficiency increases the risk of T2DM due to decreased activity of antioxidant enzymes	No funding	Low
Laouali N. et al., 2021 [22]	Prospective Cohort Study	*n* = 70,991 France Mean age at baseline = 53 years	The copper/zinc ratio was estimated from food intake, which was assessed using a validated 208-item semi-quantitative food frequency questionnaire	High Cu/Zn ratio increases T2DM risk; High zinc intake increases T2DM risk	Supported by both public and private non-profit foundations	Good
Chu A. et al., 2016 [23]	Systematic review	*n* = 334,387 Males and Females 33–84 years (range)	Dietary and serum Zn	High serum zinc levels are associated with T2DM risk	No funding	Good
Fernández-Cao J.C. et al., 2019 [1]	Systematic review, Meta-Analysis	*n* = 156,178 Males and Females 18–66 years (range)	Dietary and serum/plasma Zn	High serum zinc levels are associated with T2DM risk	No funding	Good

* Detailed assessment results are provided in the Supplementary Materials (Table S1).

**Table 5:** Characteristics of publications mentioning zinc imbalance as a consequence of T2DM.

Author, Year	Research Type	Characteristics of the Participants	Zinc Status Details	Brief Conclusions on the Problem	Funding	Quality Assessment *
Petroni M.L. et al., 2021 [24]	Review	-	Hyperzincuria	In T2DM, zinc deficiency occurs due to decreased absorption and increased excretion of the trace element from the body	No funding	Moderate
Safarzad M. et al., 2023 [19]	Case–Control Study	*n* = 112 (32 males and 80 females) Iran, Gorgan 54 ± 1.2 years (T2DM), 50.2 ± 1.7 years (control)	Serum Zn concentrations were 10,7% lower than in the control group	Serum zinc levels are lower in patients with T2DM compared to healthy controls	Supported by the Golestan University of Medical Sciences	Good
Siddiqui K. et al., 2014 [14]	Review	-	Hyperzincuria	The decrease in zinc levels in the blood occurs due to a disruption in the reabsorption of endogenous zinc during the digestion process	No funding	Low
Fernández-Cao J.C. et al., 2018 [25]	Systematic review, Meta-Analysis, Meta-Regression	*n* = 1532 Males and Females 25–75 years (range)	Lower dietary zinc intake in individuals with T2DM; plasma/whole blood zinc concentrations	The extent of serum zinc decline depends on the duration of T2DM; no difference in dietary zinc intake was found between patients and healthy individuals	No funding	Good
Fong C. et al., 2022 [15]	Umbrella Review	-	Hyperzincuria	Glucose intolerance and insulin resistance are factors in deteriorating zinc status	Supported by non-profit foundations together with an independent philanthropist	Good
Chabosseau P. et al., 2016 [26]	Review	-	Insulin content in the pancreas; serum zinc levels	Decreased zinc levels observed in pancreatic cells	Supported by several non-profit foundations	Low
Xu J. et al., 2013 [27]	Cross-Sectional Study	*n* = 239 (T2DM or prediabetes) Males and Females Median age = 55 years Age range = 20–65 years Complications: Nephropathy (*n* = 24) Retinopathy (*n* = 34) Peripheral neuropathy (*n* = 50)	Serum and Urinal Cu and Zn	Elevated Cu/Zn ratio observed in serum of patients with T2DM; Serum Zn levels decrease in the following sequence: uncomplicated T2DM -> neuropathy -> nephropathy -> retinopathy	Supported by National Science Foundation of China	Moderate

* Detailed assessment results are provided in the Supplementary Materials (Table S1).

**Table 6:** Characteristics of publications analyzing the effectiveness of zinc supplementation for the prevention and treatment of T2DM.

Author, Year	Research Type	Characteristics of the Participants	Zinc Supplement Details	Brief Conclusions on the Problem	Funding	Quality Assessment *
Wang Z. et al., 2023 [28]	Systematic review, meta-analysis	*n* = 897 Males and Females T2DM 16–65 years (range)	Zn sulfate (4–149 mg/d) Zn gluconate (4–72 mg/d) Elemental Zn (6–81 mg/d) 1–12 month	Zinc supplements (gluconate, sulfate) improve glycemic control and reduce insulin resistance	No funding	Good
Petroni M.L. et al., 2021 [24]	Review	-	No specific data	Zinc supplements improve glycemic control and reduce insulin resistance	No funding	Moderate
Heidari Seyedmahalleh M. et al., 2023 [29]	Systematic review, meta-analysis	*n* = 1067 Males and Females T2DM 46–66 years (range)	Zn sulfate (7–152 mg/d) Zn gluconate (7–120 mg/d) 6–54 weeks	Zinc supplements (gluconate, sulfate) improve lipid profile parameters	Supported by Tehran University of Medical Sciences	Good
Pompano L.M. et al., 2021 [30]	Systematic review, meta-analysis	*n* = 1042	Zn sulfate Zn gluconate Zn amino chelate 9.8–75 mg/d 4 weeks–12 months	The best therapeutic effect is achieved with long-term consumption of low doses of zinc (<25 mg/d)	Supported by the UK Government	Moderate
Wang X. et al., 2019 [31]	Systematic review, meta-analysis	*n* = 1700	Zn sulfate Zn gluconate Zn amino chelate Zn oxide Zn acetate 4–240 mg/d 1–12 months	Inorganic forms of zinc are most effective in improving glycemic control	No funding	Good
Hamedifard Z. et al., 2020 [32]	Randomized, Double-Blind, Placebo-Controlled Trial	*n* = 55 Iran Women T2DM 40–95 years (range)	Magnesium oxide 250 mg/d + Zn sulfate 150 mg/d (containing 30 mg elementary Zn) 12 weeks	Co-supplementation with zinc and magnesium improves glycemic control, lipid profile, and inflammatory markers	Supported by Research Deputy of Kashan University of Medical Sciences	Good
El Dib R. et al., 2015 [33]	Systematic review	*n* = 128 Males and females Non-diabetic adults with insulin resistance	Zn sulfate (30–200 mg/d) 4–12 weeks	Zinc supplementation has a neutral effect on glycemic control and lipid profile in residents of developed countries	No funding	Moderate
Attia J.R. et al., 2022 [34]	Randomized, Double-Blind, Placebo-Controlled Trial	*n* = 98 Australia 40–70 years (range) BMI ≥ 27 kg/m2 HbA1c of 5.7–6.4%, (39–46 mmols/mol)	Zn gluconate 30 mg/d 12 months	Zinc supplementation has no statistically significant effect on glycemic parameters	Supported by non-profit health organizations	Good
Chabosseau P. et al., 2016 [26]	Review	-	No specific data	The effect of zinc supplementation may vary depending on the presence of SLC30A8 gene polymorphisms	Supported by several non-profit foundations	Low
Kanoni S. et al., 2011 [16]	Meta-analysis	*n* = 34,150 Males and Females 11–74.8 years (range) BMI 20–29.7 kg/m2 (range)	Total Zn intake (food sources and supplements) 8.7–17.3 mg/d	Adequate zinc intake from food/supplements reduces glucose levels in patients with rs11558471 polymorphism (A)	No funding	Moderate
Nazem, M.R. et al., 2023 [35]	Randomized Placebo-Controlled Trial	*n* = 80 Males and Females 40–65 years (range) BMI 25–30 kg/m2 (range) FBG ≥ 126 mg/dL HbA1c ≥ 7	Zn gluconate 50 mg/d 8 weeks	Zinc gluconate intake lowered HbA1c, HOMA-IR, and fasting glucose levels. After the intervention, patients had a lower average BMI and an improved total antioxidant status	Supported by the Ministry of Health and Medical Education, I.R. Iran	Moderate
Lee, Y.-M. et al., 2016 [36]	Randomized, Double-Blind, Placebo-Controlled Trial	*n* = 36 Males and Females 65.0 ± 6.0 years (T2DM) 61.9 ± 7.6 years (control) BMI 30.9 ± 4.0 kg/m2 (T2DM) 30.0 ± 5.5 kg/m2 (control) HbA1c ≥ 6.5	Product prepared by the cascade fermentation of defined vegetables, fruits, and nuts and subsequently enriched with chromium (100 µg/d) and zinc (15 mg/d) 24 weeks (12+12)	A cascade-fermented dietary supplement based on fruits, nuts, and vegetables fortified with chromium and zinc did not improve glucose metabolism in patients with T2DM	Supported by Dr. Niedermaier Pharma GmbH, Hohenbrunn, Germany	Good
Asghari, S. et al., 2019 [37]	Randomized, Double-Blind, Placebo-Controlled Trial	*n* = 60 Males and Females 45.5 ± 5.4 years (T2DM, Zn) 46.2 ± 5.3 years (T2DM, control)	Zn gluconate 30 mg/d 12 weeks	Zinc gluconate intake did not lead to a significant improvement in glycemic control in the intervention group. Zinc gluconate intake partially restored adiponectin concentration in the intervention group and increased HDL levels compared to the control group	No funding	Good
Chhina, G.S. et al., 2022 [38]	Randomized, Double-Blind, Placebo-Controlled Trial	*n* = 80 Males and Females 47.62 ± 7.49 years (T2DM, Zn) 48.55 ± 8.67 years (T2DM, control) BMI 27.60 ± 4.06 kg/m2 (T2DM, Zn) 27.99 ± 3.22 kg/m2 (T2DM, control) HbA1c 7.68 ± 0.65% (T2DM, Zn) 7.57 ± 0.69% (T2DM, control)	Zn supplements (not specified) 50 mg/d in addition to metformin 12 months	Zinc supplements in combination with the hypoglycemic drug metformin reduced fasting glucose, postprandial blood glucose, and HbA1c levels, as well as improved the lipid profile compared to the control group	No funding	Low
Nani, A. et al., 2023 [39]	Randomized, Double-Blind, Placebo-Controlled Trial	*n* = 75 Males and Females 62–74 years (T2DM, Zn) 62.3–76 years (T2DM, control) BMI 24.2–30.6 kg/m2 (T2DM, Zn) 25.2–32.9 kg/m2 (T2DM, control) HbA1c 7.5–8.1% (T2DM, Zn) 7.4–8.3% (T2DM, control)	Antidiabetic drugs + two sachets per day of Eudiamet^®^, each containing myo-Ins (1950 mg), d-chiro-Ins (50 mg), α-LA (50 mg), *Gymnema sylvestre* (250 mg) and Zn (7.5 mg)	Intake of a supplement containing myo-inositol, D-chiro-inositol, α-lactalbumin, *Gymnema sylvestre*, and zinc led to an improvement in the lipid profile and a reduction in body weight	The APC was funded by Lo.Li. Pharma S.r.l., Rome, Italy	Moderate
Said, E. et al., 2020 [40]	Randomized, Double-Blind Trial	*n* = 98 Males and Females T2DM 50.2 ± 9.5 years (vit. A + E) 52.4 ± 6.8 years (vit. A + E + Zn) 50.2 ± 9.2 years (control) BMI 33.9 ± 3.7 kg/m2 (Vit. A + E) 31.9 ± 4.4 kg/m2 (Vit. A + E + Zn) 31.9 ± 3.7 kg/m2 (control)	50 000 I.U. vitamin A + 100 mg vitamin E + 75 mg Zn gluconate equivalent to 25 mg Zn 12 weeks	Zinc intake in combination with high doses of vitamins A and E improved glycemic control, β-cell function, and insulin secretion compared to the control group that consumed only vitamins A and E	No funding	Moderate
Ranasinghe, P. et al., 2018 [41]	Randomized, Double-Blind, Placebo-Controlled Trial	*n* = 200 Males and Females Prediabetes 51.9 ± 6.7 years (Zn) 51.7 ± 7.7 years (placebo) BMI 25.5 ± 3.4 kg/m2 (Zn) 24.6 ± 3.7 kg/m2 (placebo)	Zn supplements (not specified) 20 mg/d 12 months	Zinc supplementation improved glycemic control, lipid profile, and β-cell function in patients with prediabetes	Supported by National Science Foundation of Sri Lanka	Low
Karandish, M. et al., 2022 [42]	Randomized, Double-Blind, Placebo-Controlled Trial	*n* = 82 Males and Females Prediabetes 38.19 ± 4.87 years (Zn) 34.48 ± 6.45 years (Curcumin+Zn) 34.19 ± 7.03 years (placebo) BMI 29.5 ± 2.82 kg/m2 (Zn) 29.95 ± 2.56 kg/m2 (Curcumin+Zn) 30.97 ± 2.33 kg/m2 (placebo)	Zn gluconate 30 mg/d (+curcumin supplement 500 mg-BCM95) 60 days	Zinc intake in combination with curcumin had a positive effect on the lipid profile and BMI of patients with prediabetes	Supported by Ahvaz Jundishapur University of Medical Sciences	Good

* Detailed assessment results are provided in the Supplementary Materials (Table S1).

**Table 7:** Evidence-based recommendations for personalized zinc supplementation strategies in T2DM.

Personalization Criterion	Suggested Zinc Intervention ^1^	Evidence Strength ^2^	Potential Synergists	Limitations/Precautions	Key References
Zinc deficiency (<84 µg/dL in serum [87])	≥25 mg/d elemental zinc (zinc gluconate or sulfate), 12–24 weeks	High (≥ 3 RCTs and ≥ 2 meta-analyses of good and moderate quality + ≥ 3 case–controls ^3^)	Magnesium, vitamins A and E	Monitor Cu/Zn ratio; avoid prolonged high-dose use	[17,18,19,28,29,30,31,32,40]
SLC30A8 polymorphism (rs11558471 A-allele)	8.7–17.3 mg/d elemental zinc (from diet ± supplements)	Moderate (1 meta-analysis of moderate quality)	-	Genotype-specific response; requires confirmation	[16,26]
Insulin resistance/metabolic syndrome	<25 mg/d elemental zinc for ≥ 12 weeks; inorganic forms preferred	High (≥ 2 meta-analyses of good and moderate quality, 2 RCTs of good quality, 1 RCT of moderate quality)	Magnesium; myo-inositol, D-chiro-inositol, α-lactalbumin, *Gymnema sylvestre*	Ineffective if Cu/Zn > 0.55; Ineffective for residents of developed countries and large cities	[29,30,31,32,37,39]
Newly diagnosed T2DM (less than a year)	50 mg/d elemental zinc (zinc gluconate or sulfate), 12 months	Low (1 RCT, low quality)	Metformin	Zinc excess (>159 µg/dL in serum [87])	[38]
Inflammatory or oxidative complications (nephropathy, retinopathy, neuropathy)	Combination supplements (Zn + vitamins A/E)	Moderate (2 RCTs of moderate quality, 1 RCT of good quality)	Magnesium Vitamins A and E	Control oxidative-stress markers; avoid excessive fat-soluble vitamins	[27,32,35]
Normal or elevated zinc status (>159 µg/dL [87])	Additional zinc supplementation not recommended	High (2 System Reviews of good quality)	-	Risk of pro-oxidant and metabolic adverse effects	[1,23]

^1^ According to [30], high doses of zinc ≥25 mg/d and low doses of zinc < 25 mg/d. ^2^ Evidence strength rated according to the number and quality of human studies summarized in this review (AMSTAR-2, NOS, RoB 2). ^3^ Case–control studies show the risks of zinc deficiency in association with T2DM.

## Data Availability

No new data were created or analyzed in this study. Data sharing is not applicable to this article.

## Supplementary Materials

The following supporting information can be downloaded at https://www.mdpi.com/article/10.3390/diseases13120396/s1, Table S1: Risk of Bias Assessment.

## Abbreviations

The following abbreviations are used in this manuscript:

T2DM	Type 2 Diabetes Mellitus
HOMA-IR	Homeostasis Model Assessment of Insulin Resistance
HbA1c	Hemoglobin A1C
ROS	Reactive Oxygen Species
RCT	Randomized Controlled Trial
LDL	Low-Density Lipoprotein
HDL	High-Density Lipoprotein
TC	Total Cholesterol
TG	Triglyceride
CVD	Cardiovascular Disease
BMI	Body Mass Index
NOS	Newcastle–Ottawa Scale
